# Phylogeographic analysis of *Siraitia grosvenorii* in subtropical China provides insights into the origin of cultivated monk fruit and conservation of genetic resources

**DOI:** 10.1002/ece3.10181

**Published:** 2023-06-09

**Authors:** Bingbin Xie, Bowen Lai, Liping Chen, Sujuan Wei, Shaoqing Tang

**Affiliations:** ^1^ Key Laboratory of Ecology of Rare and Endangered Species and Environmental Protection, Ministry of Education Guangxi Normal University Guilin China; ^2^ Guangxi Key Laboratory of Rare and Endangered Animal Ecology, College of Life Science Guangxi Normal University Guilin China

**Keywords:** chloroplast DNA, orthologous nuclear gene, phylogeography, population structure, *Siraitia grosvenorii*

## Abstract

*Siraitia grosvenorii*, an economically important plant species with high medicinal value, is endemic to subtropical China. To determine the population structure and origin of cultivated *S. grosvenorii*, we examined the variation in three chloroplast DNA regions (*trn*R*‐atp*A, *trn*H*‐psb*A, *trn*L*‐trn*F) and two orthologous nuclear genes (*CHS* and *EDL2*) of *S. grosvenorii* in 130 wild individuals (selected from 13 wild populations across its natural distribution range) and 21 cultivated individuals using a phylogeographic approach. The results showed three distinct chloroplast lineages, which were restricted to different mountain ranges, and strong plastid phylogeographic structure. Our findings suggest that *S. grosvenorii* likely experienced ancient range expansion and survived in multiple refuges in subtropical China during glacial periods, resulting in population fragmentation in different mountainous areas. Our results also demonstrated that wild populations in Guilin (Guangxi, China) share the same gene pool as cultivated *S. grosvenorii*, suggesting that current cultivars were collected directly from local wild resources, consistent with the principles of “nearby domestication.” The results of this study provide insights into improving the efficiency of *S. grosvenorii* breeding using a genetic approach and outline measures for the conservation of its genetic resources.

## Graphical abstract

The manuscript presents the first phylogeographic analysis of *Siraitia grosvenorii*, an economic plant species distributed in subtropical China. Our results clarify not only the population genetic structure of *S. grosvenorii*, but also determin the geographic origins of cultivated *S. grosvenorii*. The current study provide important information for the utilization and conservation of the valuable genetic resources of *S. grosvenorii*.

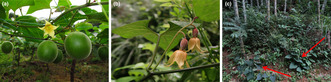

## 1 INTRODUCTION

**FIGURE 1:**
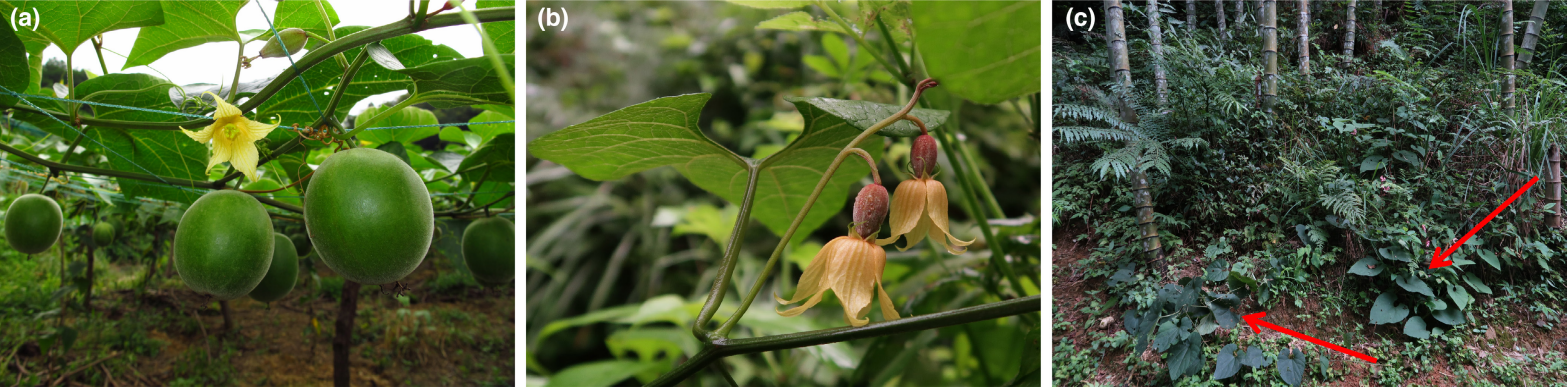
Pictures of cultivated and wild *S. grosvenorii* individuals and the habitat of this species. (a) Cultivated individual; (b) wild individual; (c) habitat.

*Siraitia grosvenorii*, also known as monk fruit or Luohanguo, is a dioecious perennial vine belonging to the *Siraitia* genus of the Cucurbitaceae family (Figure 1). The genus *Siraitia* comprises four species, among which only *S. grosvenorii* is native to China (Li, 1993). The close relationship between *S. grosvenorii* and *Siraitia siamensis* is supported by morphological similarities and adjacent distribution in China. However, the seed of *S. grosvenorii* is typically 2‐layered winged, which is easily distinguishable from that of *S. siamensis*.

*Siraitia grosvenorii* has been cultivated for more than 100 years in China (Ding et al., 2015). Currently, *S. grosvenorii* is prevalent in the Yongfu, Longsheng, and Lingui counties of Guilin (Guangxi, China), and is considered an economically important crop in Guangxi. *S. grosvenorii* was introduced from Guangxi into other provinces in southern China. In addition to its use as Chinese traditional medicine (Chinese Pharmacopoeia Commission, 2015), *S. grosvenorii* is highly valued in the food industry. The fruit of *S. grosvenorii* contains mogrosides, which are approximately 300 times sweeter than sucrose (Kasai et al., 1989). Because mogrosides are natural and low‐calorie compounds, the mogroside extract from *S. grosvenorii* is considered to be an ideal sugar substitute (Yan et al., 2008). Given the increasing demand for non‐nutritive sweeteners from natural sources, *S. grosvenorii* sweeteners have been widely used in the food and beverage industries for the development of low‐calorie products (Pandey & Chauhan, 2019). In 2020, *S. grosvenorii* was planted in an area of over 10,000 ha in Guilin, accounting for more than 85% of the global production (Lu et al., 2022). However, because of the continuous vegetative propagation of *S. grosvenorii*, its cultivated accessions have become more vulnerable to pests and diseases (Li et al., 2004). Wild genetic resources allow plant breeders to select and breed for the desired characteristics, which facilitates not only the maintenance but also the improvement of agricultural productivity (Day‐Rubenstein et al., 2005; Prescott‐Allen & Prescott‐Allen, 2013). To fully utilize the genetic resources of *S. grosvenorii*, a proper understanding of the population structure of its wild accessions and the origin of its cultivated accessions is greatly needed.

The area of China ranging from 22°N to 34°N and bordered by 105°E in the west to the coastline in the east is generally referred to as subtropical China (Zhao et al., 1986). This area is characterized by a complex landform, with numerous temperate forests at high elevations and extensive tropical and subtropical forests at lower altitudes (Qian & Ricklefs, 2000). *S. grosvenorii* is naturally distributed in subtropical China and mainly grows in the Nanling (comprising a series of five connected mountain ranges spanning from west to east, including Yuechengling, Dupangling, Mengzhuling, Qitianling, and Dayuling), Dayaoshan, Yunkai, Jiulianshan, and Wugongshan Mountains at an altitude of 400–1400 meters. Global climatic fluctuations, particularly Quaternary climate oscillations, might have profoundly shaped the distribution range and genetic structure of plants (Abbott et al., 2000; Avise, 2000). Although glaciers are not found at low elevations (<2500 m) in subtropical China (Ni et al., 2010), a cold and dry climate has developed in this region because of the intensification of winter monsoons (Shi et al., 2006; Wang et al., 2012). *S. grosvenorii* diverged from members of the Cucurbitaceae family approximately 40–60 million years ago (Mya) (Guo et al., 2020; Xia et al., 2018), and its current genetic structure and diversity across its distributional range might be influenced by the Quaternary climate changes. Previously, the genetic diversity of the wild populations and cultivars of *S. grosvenorii* was analyzed using random amplified polymorphic DNA (RAPD), inter‐simple sequence repeat (ISSR), and amplified fragment length polymorphism (AFLP) markers, which revealed a high level of genetic differentiation among the wild populations (Tang, Bin, et al., 2007). Additionally, the genetic diversity of cultivars was discovered to be much lower than that of wild populations (Peng et al., 2005; Tang, Bin, et al., 2007; Zhou et al., 2005; Zhou & Tang, 2006). Based on RAPD markers, Tang, Li, et al. (2007) speculated that clonal growth plays a role in shaping the spatial genetic structure of *S. grosvenorii*. However, the population genetic structure of *S. grosvenorii* at different geographical scales and its evolutionary history, such as potential refugia and population expansion, remains unknown, mainly because the DNA markers used in previous studies offer limited utility in inferring population structure and dynamics.

In this study, we report the results of the phylogeographical analysis of *S. grosvenorii* in subtropical China using three chloroplast DNA (cpDNA) fragments and two orthologous nuclear genes. We aimed to (1) investigate the genetic structure of *S. grosvenorii*, and infer its main potential refugial locations and demographical history, and (2) determine the geographical origin and ancestral populations of cultivated *S. grosvenorii*. Information on the population structure and demographical history of *S. grosvenorii* is important for the genetic improvement of its cultivars and the conservation of its wild germplasm.

## 2 MATERIALS AND METHODS

### 2.1 Sampling

A total of 151 individuals of *S. grosvenorii* were collected in this study, including 130 individuals selected from 13 wild populations (10 individuals per population), 4 cultivated male individuals, and 17 female individuals from 7 cultivated accessions (at least 1 individual per accession). Details of the sampling are shown in Table 1. Because *S. grosvenorii* exhibits clonal reproduction and has a small population size, the individual plants were sampled from sites located >10 m apart (An et al., 2015; Gong et al., 2016; Zhang et al., 2022). Fresh leaves were dried in silica gel for subsequent DNA extraction. G. L. Shi 14316 (IBSC0208492!) was serve as voucher specimen for the population ZQ because they were collected in the same location. The voucher specimens of remaining populations were deposited in the Herbarium of the Guangxi Institute of Botany (IBK), China.Voucher specimen numbers are listed in Table 1.

**TABLE 1:** Details of the sampling and voucher specimen numbers of *S. grosvenorii.*

Population code/ accession	Latitude (N)/longitude (E)	Altitude (m)	Simple size	Voucher specimen number
MES	25°52′/110°24′	700	10	IBK00446215
BL	24°47′/110°05′	550	10	IBK00446218
SJ	25°40′/109°41′	400	10	IBK00446216
JT	25°26′/109°50′	500	10	–
JX	24°06′/110°15′	850	10	IBK00446655
DX	25°14′/111°47′	719	10	IBK00445176
HZ	24°37′/111°33′	600	10	IBK00446217
ZQ	23°36′/112°30′	347	10	IBSC0208492
RY	24°52′/113°05′	900	10	IBK00446651
SG	24°42′/113°49′	400	10	IBK00445175
JLS	24°37′/114°29′	443	10	IBK00445173
WGS	27°29′/114°15′	428	10	IBK00445174
PB	22°27′/109°49′	850	10	IBK00446649
Wild populations			130	
Cultivated male plants			4	IBK00446648
Yiyuanshengwuzi			1	
Jinyou			1	
Jifusi			1	
Funong			1	
Qingpiguo			6	IBK00446219
Hongmaoguo			5	
Baopeng			2	
Cultivar			21	
Total			151	

*Note*: MES, BL, SJ, JT, JX, DX, HZ, ZQ, RY, SG, JLS, WGS, and PB represent wild populations. Yiyuanshengwuzi, Jinyou, Jifusi, Funong, Qingpiguo, Hongmaoguo, and Baopeng represent cultivated female accessions. “–” represent destroyed. Voucher specimen (IBK00446219) is available for all cultivated female accessions.

### 2.2 DNA extraction, PCR amplification, and DNA sequencing

Genomic DNA was extracted from the dried leaves using the modified cetyltrimethylammonium bromide (CTAB) method (Doyle & Doyle, 1987); the modifications to the CTAB method included a 2‐h incubation in 65°C water bath, and two rounds of DNA extraction using chloroform‐isoamyl alcohol (24: 1). Three highly polymorphic cpDNA fragments, including *trn*R*‐atp*A (Dane & Lang, 2004), *trn*H*‐psb*A (Sang et al., 1997), and *trn*L*‐trn*F (Taberlet et al., 1991) were selected. However, the universal nuclear marker, nrITS, could not be applied to *S. grosvenorii* as we failed to sequence the PCR products. Therefore, two nuclear genes, *chalcone synthase* (*CHS*) and *EID1‐like F‐box protein 2* (*EDL2*), were selected. *CHS* has been used in phylogeographic studies (Ikeda et al., 2009; Liao et al., 2010; Wang et al., 2018). The nucleotide sequence of the *EDL2* gene of *Momordica charantia* (Cucurbitaceae) was obtained from the National Center for Biotechnology Information (NCBI; GenBank accession no. XR_002601804), and its orthologs were identified using the OrthoMCL database (http://orthomcl.org/orthomcl/). The nuclear genes were PCR amplified with sequence‐specific primers designed using Primer3Web (http://primer3.wi.mit.edu/). Primer details are given in Table 2. The amplification was carried out in 50‐μL reactions, each containing 0.5 μL of genomic DNA, 5 μL of 10× PCR buffer (Mg^2+^ plus), 4 μL of dNTP mix (2.5 μM), 0.5 μL of each primer (50 mM), and 2.5 U of ExTaq DNA polymerase (TaKaRa, Dalian, China). The amplification program was as follows: predenaturation at 94°C for 5 min, followed by 30 cycles of denaturation at 94°C for 30 s, annealing at 55 or 58°C (Table 2) for 30 s, and elongation at 72°C for 1 min, and a final extension at 72°C for 10 min. After purification of PCR products, Sanger sequencing was conducted by Sangon Biotech (Shanghai, China).

**TABLE 2:** Primers used in this study for DNA amplification and sequencing.

Target DNA	Primer sequence (5′ → 3′)	Reference	Tm (°C)
cpDNA			
*trn*R*‐atp*A	F: AGGTTCAAATCCTATTGGACGCA	Dane and Lang (2004)	55
	R: TTTTGAAAGAAGCTATTCARGAAC		
*trn*H*‐psb*A	F: GTTATGCATGAACGTAATGCTC	Sang et al. (1997)	55
	R: CGCGCATGGTGGATTCACAATCC		
*trn*L*‐trn*F	F: GGTTCAAGTCCCTCTATCCC	Taberlet et al. (1991)	55
	R: ATTTGAACTGGTGACACGAG		
Nuclear genes			
*CHS*	F: GCCACCCGTCTTATTAGCCA	This study	58
	R: TGACGCGCTGTGTGTGTGCACACACACCC		
*EDL2*	F: AAAGGGRCAYCTYAGTGAG	This study	55
	R: AACTCRGAYGTCTCYTCAG		

*Note*: Tm—melting temperature of primer pair.

Abbreviation: cpDNA, chloroplast DNA.

Genes with double peaks at polymorphic sites were regarded as heterozygous. Haplotypes were determined using the method described by Clark (1990) if the gene sequence contained a single heterozygous site, and through the cloning and sequencing of PCR products if the sequence contained two or more peaks. To clone the gene, DNA was amplified using high‐fidelity PrimeSTAR HS DNA polymerase (TaKaRa, Dalian, China), as described above. The cloned DNA was purified and subjected to Sanger sequencing by Sangon Biotech (Shanghai, China); 10 clones were sequenced per PCR product. All haplotype sequences in this study were deposited in the GenBank database under accession numbers listed in Tables 3, 4, 5, 6.

**TABLE 3:** Variable sites and GenBank accession numbers of seven haplotypes of *S. grosvenorii* based on the sequence of *trn*R*‐atp*A cpDNA spacers.

Chlorotype	Nucleotide position																									GeneBank accession number
	37	46	102	232	291	292	367	418	456	474	540	541	542	543	556	557	561	564	569	573	574	576	580	581	602	
C1	C	#	T	A	T	A	G	G	−	−	A	T	T	T	T	T	T	−	−	−	−	﹡	A	T	T	MK357026
C2	A	−	A	C	A	T	T	A	$	C	.	.	.	.	A	A	A	−	−	−	−	−	T	.	.	MK357027
C3	A	−	A	C	.	.	T	A	$	G	.	.	.	.	A	A	A	−	−	−	−	−	T	.	.	MK357028
C4	.	−	.	.	.	.	.	.	−	−	.	.	.	.	.	.	.	&	⊕	A	+	﹡	A	T	.	MK357029
C5	.	−	.	.	.	.	T	.	−	−	.	A	A	A	A	A	A	−	−	−	−	−	−	−	G	MK357030
C6	A	−	A	C	.	.	T	A	$	G	T	.	.	.	A	A	A	−	−	−	−	−	T	.	.	MK357031
C7	.	−	.	.	.	.	.	.	−	−	.	.	.	.	.	.	.	−	⊕	T	+	﹡	A	T	.	OQ134869

*Note*: “.”: Character states are the same as C1; “–”: Absence; “#”: TTATAATATAATAA; “$”: CTAATAAATGAAAACATA; “&”: AATTA; “⊕”: TAAT; “+”: AT; “﹡”: AATA.

**TABLE 4:** Variable sites and GenBank accession numbers of seven haplotypes of *S. grosvenorii* based on the sequence of *trn*H*‐psb*A and *trn*L*‐trn*F cpDNA spacers.

Chlorotype	*trn*H*‐psb*A				*trn*L*‐trn*F				
	Nucleotide position			GeneBank accession number	Nucleotide position				GeneBank accession number
	726	742	806		906	974	1000	1159	
C1	T	T	C	MK356960	T	T	G	C	MK357032
C2	C	C	.	MK356961	G	A	C	.	MK357033
C3	C	C	.	MK356962	G	A	C	T	MK357034
C4	.	.	.	MK356963	.	.	.	.	MK357035
C5	C	C	.	MK356964	.	A	C	.	MK357036
C6	C	C	T	MK356965	G	A	C	T	MK357037
C7	.	.	.	MK356963	.	.	.	.	MK357035

*Note*: “.”: Character states are the same as C1.

**TABLE 5:** Variable sites and GenBank accession numbers of 45 haplotypes of *S. grosvenorii* based on the sequence of *CHS*.

Haplotype	Nucleotide position																									GeneBank accession number
	5	35	54	88	128	155	213	249	267	318	330	342	384	417	444	480	527	532	755	780	792	846	906	993	999	
H1	C	C	T	C	A	C	C	T	A	C	C	C	G	C	C	T	C	G	A	A	G	G	G	A	G	MK356966
H2	.	.	C	.	.	.	.	C	.	.	.	.	.	.	.	.	.	A	.	.	.	.	A	G	.	MK356967
H3	.	.	.	.	.	.	.	C	.	.	.	.	.	.	.	.	.	.	.	.	.	.	.	.	.	OQ134870
H4	.	.	C	.	.	.	.	C	.	.	.	.	.	.	.	.	.	.	.	.	.	.	A	G	.	MK356969
H5	.	.	.	.	T	.	.	.	.	.	.	A	.	.	.	.	.	.	.	.	.	.	.	.	.	MK356970
H6	.	.	.	.	.	.	.	C	.	.	.	.	.	.	.	.	.	A	.	.	.	.	A	G	.	OQ134871
H7	.	.	.	.	T	.	.	.	.	.	.	.	.	.	.	.	.	.	.	.	.	.	.	.	.	MK356972
H8	.	.	C	.	.	.	.	C	.	.	.	.	.	.	.	.	.	.	.	.	.	.	A	.	.	MK356973
H9	.	.	.	.	T	.	.	.	.	.	.	A	.	.	.	.	.	.	.	.	.	.	.	G	.	MK356974
H10	.	.	C	.	.	.	.	.	.	.	.	.	.	.	.	.	.	A	.	.	.	.	A	G	.	MK356975
H11	.	.	.	.	.	.	.	.	G	.	.	.	.	.	.	.	.	A	.	.	.	A	.	.	.	MK356976
H12	.	.	.	.	.	.	.	.	.	.	.	A	.	.	.	.	.	.	.	.	.	.	.	.	.	MK356977
H13	.	.	.	.	.	.	.	.	.	.	.	A	.	.	.	C	.	A	.	.	.	A	.	G	.	MK356978
H14	.	.	.	.	.	.	.	.	G	.	.	.	.	.	.	.	.	A	.	.	.	A	.	G	.	MK356979
H15	.	.	.	.	.	.	.	C	.	.	.	.	T	.	.	.	.	.	.	.	.	.	.	.	.	MK356980
H16	.	.	.	.	.	.	.	.	.	.	.	A	.	.	.	.	A	.	.	.	.	.	.	.	.	MK356981
H17	.	.	.	.	.	G	.	.	.	.	.	A	.	.	.	.	.	.	.	.	A	.	.	.	.	MK356982
H18	.	.	.	.	.	.	.	.	.	.	.	.	.	T	.	C	.	A	.	.	.	A	.	G	.	MK356983
H19	.	.	.	.	.	.	.	.	.	.	.	A	.	.	.	.	.	.	.	.	.	.	.	G	.	MK356984
H20	.	.	.	.	.	.	.	.	.	.	.	.	.	T	G	.	.	A	.	.	.	A	.	G	.	MK356985
H21	.	.	.	.	.	.	.	.	.	.	.	.	.	.	.	.	.	A	.	.	.	.	.	G	.	MK356986
H22	.	.	.	.	.	.	.	.	.	.	.	.	.	.	.	.	.	.	.	.	.	.	.	G	.	MK356987
H23	.	.	.	.	.	G	.	.	.	.	.	A	.	.	.	.	.	.	.	.	.	.	.	.	.	MK356988
H24	.	.	.	.	.	.	.	.	.	.	.	.	.	T	G	C	.	A	.	.	.	A	.	G	.	MK356989
H25	.	.	.	.	.	.	.	.	.	.	.	.	.	.	.	.	.	A	.	G	.	A	.	G	.	MK356990
H26	.	.	.	.	.	.	.	.	.	.	.	.	.	.	.	.	.	A	.	G	A	.	.	G	.	MK356991
H27	.	.	.	.	.	G	.	.	.	.	.	A	.	.	.	.	.	.	.	.	.	A	.	.	.	MK356992
H28	.	.	.	.	.	.	.	.	.	.	.	.	.	T	G	C	.	A	.	.	.	A	.	.	.	MK356993
H29	.	.	.	.	.	.	.	.	.	.	.	.	.	.	.	.	.	A	.	.	.	.	.	.	.	MK356994
H30	.	.	C	.	.	.	.	.	.	.	.	A	.	.	.	.	.	.	.	.	.	.	.	G	.	MK356995
H31	.	.	C	.	.	.	.	C	.	.	.	.	.	.	.	.	.	A	.	.	.	.	A	.	.	MK356996
H32	.	.	.	.	.	.	.	.	.	.	.	.	.	T	.	C	.	.	.	.	.	A	.	G	.	MK356997
H33	.	.	.	.	.	.	.	.	.	.	.	.	.	T	G	C	.	.	.	.	.	A	.	G	.	MK356998
H34	.	.	.	.	.	.	A	.	.	.	.	A	.	.	.	.	.	.	.	.	.	.	.	.	.	MK356999
H35	.	.	.	T	.	.	.	.	.	.	.	A	.	.	.	.	.	.	.	.	.	.	.	.	.	MK357000
H36	.	.	.	.	.	.	.	.	.	.	.	.	.	T	G	C	.	A	G	.	.	.	A	G	.	MK357001
H37	.	.	.	.	.	.	.	.	.	.	.	.	.	T	.	.	.	A	.	G	.	A	.	G	.	MK357002
H38	.	.	.	.	.	.	.	.	.	.	.	.	.	T	G	C	.	A	G	.	.	.	.	G	.	MK357003
H39	.	.	.	.	.	.	.	.	.	T	.	A	.	.	.	.	.	.	.	.	.	A	.	.	.	MK357004
H40	.	.	.	.	.	.	.	.	.	.	.	.	.	.	.	.	.	A	.	.	.	A	.	G	.	MK357005
H41	.	.	.	.	.	.	.	.	.	.	.	.	.	T	G	C	.	A	G	.	.	A	.	G	.	MK357006
H42	.	G	.	.	.	.	.	.	.	.	T	.	.	T	G	C	.	.	.	.	.	A	.	G	.	MK357007
H43	.	.	.	.	.	.	.	.	.	.	.	A	.	.	.	.	.	.	.	.	.	A	.	G	.	MK357008
H44	T	.	.	.	.	.	.	.	.	.	.	A	.	.	.	.	.	.	.	.	.	.	.	.	.	MK357009
H45	.	.	.	.	.	.	.	.	G	.	.	.	.	.	.	.	.	A	.	.	.	A	.	G	A	MK357010

*Note*: “.”: Character states are the same as H1.

**TABLE 6:** Variable sites and GenBank accession numbers of 15 haplotypes of *S. grosvenorii* based on the sequence of *EDL2*.

Haplotype	Nucleotide position													GeneBank accession number
	22	44	109	175	196	199	208	271	310	376	418	476	520	
E1	A	G	G	G	C	C	T	T	T	A	A	C	A	MK357011
E2	.	.	.	.	.	.	.	.	.	.	.	.	C	MK357012
E3	.	.	.	.	.	.	.	.	.	.	G	.	C	MK357013
E4	.	.	.	.	.	.	.	.	.	.	G	.	.	MK357014
E5	.	.	.	.	.	.	.	.	.	.	.	T	C	MK357015
E6	.	.	.	A	.	.	.	.	.	.	G	.	C	MK357016
E7	T	.	.	.	.	.	.	.	.	.	G	.	C	MK357017
E8	.	.	.	.	G	T	.	.	.	.	G	.	C	MK357018
E9	.	.	.	.	.	.	.	.	C	.	G	.	C	MK357019
E10	.	T	.	.	.	.	.	.	.	.	G	.	C	MK357020
E11	.	.	.	.	.	.	A	.	.	.	.	.	C	MK357021
E12	.	.	.	.	.	.	.	C	.	.	G	.	C	MK357022
E13	.	.	.	.	.	.	.	.	C	G	G	.	C	MK357023
E14	.	.	A	.	.	.	.	.	.	.	G	.	C	MK357024
E15	T	.	.	A	.	.	.	.	.	.	G	.	C	MK357025

*Note*: “.”: Character states are the same as E1.

### 2.3 Sequence analysis

To analyze cpDNA and nuclear data, DNA sequences were aligned using MUSCLE 3.8.31 (Thompson et al., 1994) in MEGA 7.0.14 (Kumar et al., 2016), and edited manually in BioEdit 7.0.1 (Hall, 1999) where necessary. Three cpDNA regions (*trn*R‐*atp*A, *trn*H‐*psb*A, and *trn*L‐*trn*F) were amplified from 151 individuals and aligned individually, then they were concatenated using the concatenate sequence plugin in PhyloSuite 1.2.2 (Zhang et al., 2020) to define cpDNA haplotypes and to conduct further analysis. The nuclear DNA sequences of *CHS* and *ELD2* were analyzed independently.

The total and population‐specific numbers of haplotypes, haplotype diversity (*h*), and nucleotide diversity (*π*) were calculated using DNAsp 5.0 (Librado & Rozas, 2009). The haplotype network maps of cpDNA regions (*trn*R‐*atp*A, *trn*H‐*psb*A, and *trn*L‐*trn*F) and nuclear genes (*CHS* and *EDL2*) were constructed using Network 5.0 (Bandelt et al., 1999) with the median‐joining method using the maximum parsimony (MP) approach. Insertions and deletions (indels) were treated as a single mutational event (Caicedo & Schaal, 2004). A map depicting the geographic distribution of haplotypes in each population was drawn using ArcMap GIS 10.2 (ESRI, Redlands, CA, USA).

A phylogenetic tree of cpDNA haplotypes was constructed using the maximum likelihood (ML) method and implemented with the IQ‐TREE 1.6.12 program (Nguyen et al., 2015). The sister taxon *Siraitia siamensis* (GenBank accession no. MK75585) was selected as the outgroup. The F81 + F model was selected as the most appropriate substitution model based on Bayesian information criterion (BIC) implemented in IQ‐TREE ModelFinder (Kalyaanamoorthy et al., 2017). Statistical support for branching patterns was estimated using 1000 bootstrap replicates.

Values of total genetic diversity (*H*_T_) and within‐population genetic diversity (*H*_S_) were calculated using PERMUT 2.0 (Pons & Petit, 1996). To infer the phylogeographic structure of *S. grosvenorii*, significant differences between *G*_ST_ and *N*_ST_ were calculated using a permutation test in PERMUT 2.0 with 1000 permutations. Analyses of molecular variance (AMOVA) were performed using Arlequin 3.11 (Excoffier et al., 2005), and the significance level of each variance component was assessed using 1000 permutations. In addition, AMOVA analyses with three chlorotype‐based genetic lineages identified in this study were also conducted. Genetic variation was quantified at three hierarchical levels: among populations, within populations, and among lineages of populations identified by three chlorotype‐based genetic lineages found in this study.

Divergence times for the three chlorotype‐based lineages, identified based on cpDNA data, were estimated using BEAST 1.6.1 (Drummond & Rambaut, 2007). A total of 1.0 × 10^−9^ (minimum) to 3.0 × 10^−9^ (maximum) substitutions per site per year was used as a range of average mutation rates, as reported for synonymous sites in the chloroplast genes of angiosperm species (Wolfe et al., 1987). The strict molecular clock model was used as the basic model for branch rates implemented in BEAST (Zuckerkandl & Pauling, 1965), which can be calibrated by setting a substitution rate or dates of specific nodes or node sets (Drummond & Rambaut, 2007). In this study, we used the strict molecular clock model and the GTR + G + I model with four Gamma categories. The GTR + G + I model was selected by the ModelFinder (Kalyaanamoorthy et al., 2017) plugin in PhyloSuite 1.2.2, with the setting of “model for BEAST1.” We chose the Yule speciation prior and Markov chain Monte Carlo (MCMC) with 60 million generations and sampling every 1000 generations. Tracer 1.7 (Rambaut et al., 2018) was used to check whether the parameters converged and the effective sample size (ESS) exceeded 200, indicating acceptable mixing and sufficient sampling. The maximum clade credibility (MCC) tree was summarized using TreeAnnotator 1.6.1 (BEAST package). The resulting chronograms were visualized in FigTree 1.4.0 (http://tree.bio.ed.ac.uk/software/figtree/).

The historical population dynamics of *S. grosvenorii* was determined by performing neutrality tests and estimating the mismatch distribution of 13 wild populations and three chlorotype‐based genetic lineages identified in this study. Neutrality tests were performed by calculating Tajima's *D* (Tajima, 1989) and Fu and Li's *D* (Fu & Li, 1993) using DNAsp 5.0, and Fu's *F*s (Fu, 1997) using Arlequin 3.11. Mismatch distribution analyses were performed using a sudden (stepwise) expansion model (Rogers & Harpending, 1992) in DNAsp 5.0. The goodness‐of‐fit between observed and expected mismatch distributions was tested by calculating the sum of squared deviations (SSD) and raggedness index (*H*_Rag_) using 1000 parametric bootstrap replicates in Arlequin 3.11. All tests performed in Arlequin 3.11 and DNAsp 5.0 used default settings.

## 3 RESULTS

### 3.1 Population structure based on chlorotypes

**FIGURE 2:**
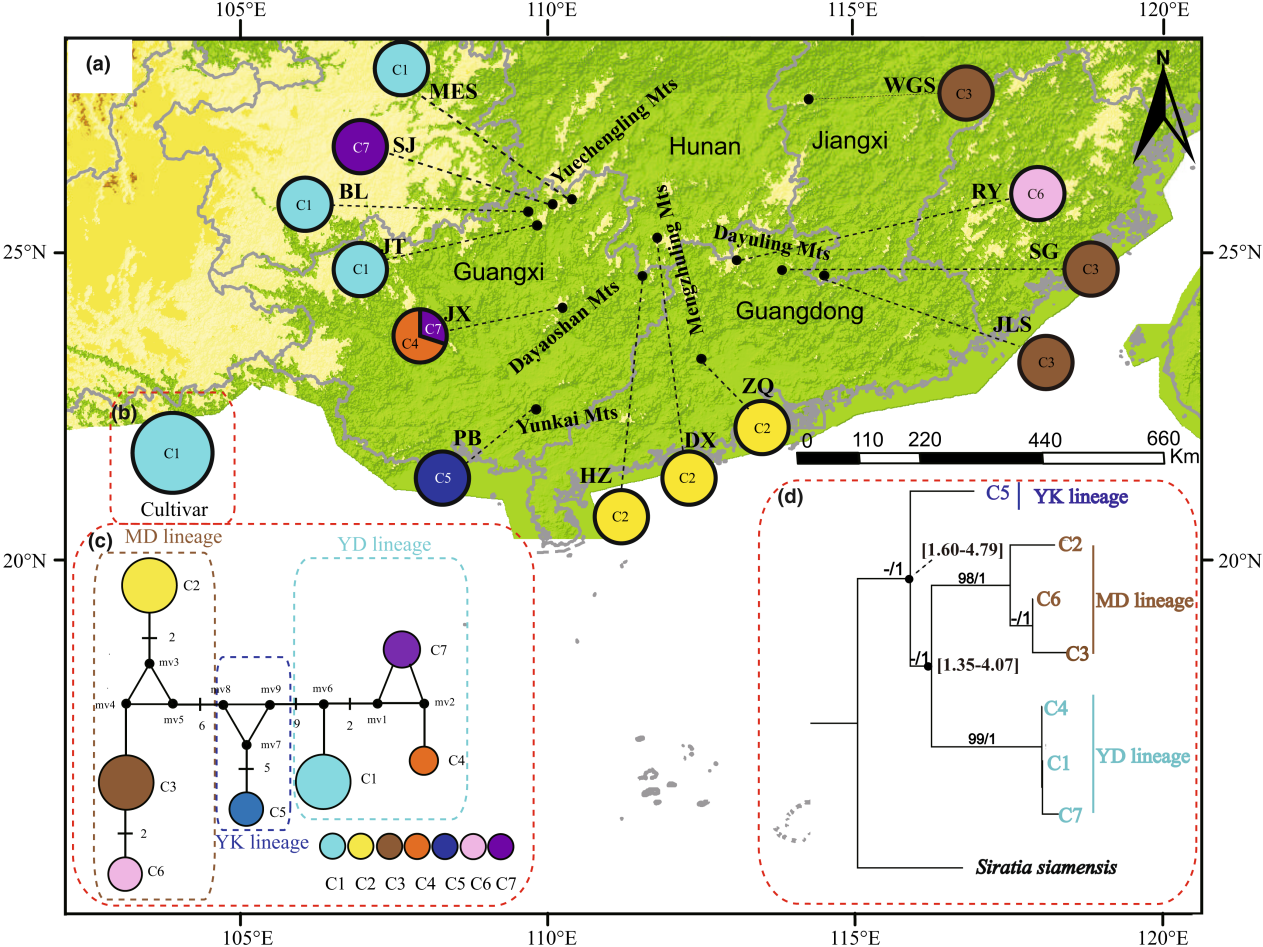
Geographical distribution, median‐joining network, and phylogenetic tree of seven chlorotypes of *S. grosvenorii*. (a) Chlorotypes of 13 wild populations of *S. grosvenorii*. Each color represents a chlorotype. (b) Chlorotypes of 21 individuals of cultivated *S. grosvenorii*. (c) Median‐joining network of seven chlorotypes resolved in *S. grosvenorii*. Small solid circles with “mv” indicate unsampled or extinct haplotypes. Each line between haplotypes without short black bars represents a mutational step. Short black bars between haplotypes represent multiple changes (indicated by adjacent numbers). MD, YD, and YK represent the three genetic lineages identified by the phylogenetic analysis of chlorotypes. (d) Maximum likelihood (ML) phylogenetic tree and maximum clade credibility (MCC) tree of the seven chlorotypes (C1–C7) detected in *S. grosvenorii* using *S. siamensis* as an outgroup. Numbers above the branches indicate the result of ML bootstrap (BP) analysis (no numbers correspond to supports weaker than 80% BP) and posterior probability (PP) of the MCC tree. The node age (million years ago [Mya]) of major lineages is indicated next to each node, based on the minimum and maximum rates of synonymous substitutions in cpDNA.

The alignment of the combined cpDNA sequences was 1259 bp in length, and contained 32 polymorphic sites, resulting in seven chlorotypes (C1–C7; Tables 3 and 4). The different chlorotypes and their network and geographical distribution are presented in Table 7 and Figure 2a,c. Most populations contained only one chlorotype, and only the JX population contained two chorotypes (C4, C7). Thus, within‐population diversity was observed only in the JX population (Table 7). The *h* and *π* values of wild populations were 0.822 and 0.00978, respectively (Table 7). No ancestral haplotype was found in the chlorotype network. C1 was the most abundant chlorotype, and was shared by three populations (MES, BL, and JT) and cultivars. Two chlorotypes were shared by sets of three different populations: chlorotype C2 was shared by HZ, DX, and ZQ, and chlorotype C3 by SG, JLS, and WGS. Chlorotypes C4, C5, and C6 were unique to populations JX, PB, and RY, respectively. Chlorotype C7 was shared by populations SJ and JX. In the chlorotype network (Figure 2c), C1 was separated from C4 and C7 by five and four mutational steps, respectively, and separated from C5 by 16 mutational steps. The other part of the network contained three chlorotypes (C2, C3, and C6), which were found in seven populations (HZ, DX, ZQ, SG, JLS, WGS, and RY). Chlorotype C2 was separated from C3 by four mutational steps, and C3 was separated from C6 by two mutational steps.

**TABLE 7:** Genetic diversity and haplotype distribution of *S. grosvenorii*, based on cpDNA and nuclear gene (*CHS*, *EDL2*) sequences.

Wild population/cultivar	cpDNA			*CHS*			*EDL2*		
	Chlorotype	*h*	π	Haplotype	*h*	π	Haplotype	*h*	π
MES	C1(10)	0	0	H5(2), H12(11), H24(2), H30(1), H31(2), H32(1), H33(1)	0.695	0.00316	E2(1), E3(7), E12(12)	0.542	0.00100
BL	C1(10)	0	0	H5(20)	0	0	E1(9), E3(10), E4(1)	0.574	0.00305
SJ	C7(10)	0	0	H5(18), H12(2)	0.189	0.00018	E1(10), E3(5), E12(5)	0.658	0.00238
JT	C1(10)	0	0	H1(2), H2(16), H6(1), H29(1)	0.363	0.00128	E2(10), E3(10)	0.526	0.00087
JX	C4(7), C7(3)	0.467	0.00077	H3(20)	0.479	0.00046	E2(1), E3(1), E8(2), E10(8), E11(8)	0.700	0.00315
YD lineage		0.564	0.00187		0.763	0.00303		0.806	0.00240
DX	C2(10)	0	0	H11(2), H12(14), H13(3), H14(1)	0.500	0.00195	E3(2), E5(8), E6(8), E7(2)	0.695	0.00281
HZ	C2(10)	0	0	H3(3), H12(11), H15(5), H16(1)	0.642	0.00145	E1(2), E5(16), E8(2)	0.358	0.00180
ZQ	C2(10)	0	0	H14(1), H24(9), H45(10)	0.574	0.00251	E3(7), E6(7), E7(3), E15(3)	0.747	0.00160
RY	C6(10)	0	0	H21(1), H25(6), H36(7), H37(1), H38(1), H39(1), H40(2), H41(1)	0.805	0.00382	E2(10), E3(10)	0.526	0.00087
SG	C3(10)	0	0	H12(3), H17(5), H18(7), H23(2), H24(1), H42(1), H43(1)	0.816	0.00422	E3(9), E9(8), E13(2), E14(1)	0.658	0.00134
JLS	C3(10)	0	0	H17(3), H18(1), H19(1), H20(3), H21(1), H22(1), H23(4), H24(2), H25(1)，H26(1), H27(1), H28(1)	0.932	0.00421	E3(5), E5(6), E6(3), E9(6)	0.774	0.00263
WGS	C3(10)	0	0	H12(3), H19(2), H25(7), H40(2), H44(6)	0.784	0.00289	E9(20)	0	0
MD lineage		0.621	0.00204		0.919	0.00440		0.822	0.00267
PB	C5(10)	0	0	H12(12), H34(7), H35(1)	0.726	0.00098	E2(3), E3(11), E6(6)	0.616	0.00117
YK lineage		0	0		0.726	0.00098		0.616	0.00117
Wild populations		0.822	0.00978		0.909	0.00406		0.854	0.00264
Cultivars	C1(21)	0	0	H1(4), H2(19), H3(2), H4(2), H5(7), H6(4), H7(1), H8(1), H9(1), H10(1)	0.761	0.00295	E1(14), E2(4), E3(24)	0.567	0.00158
Total		0.794	0.00972		0.914	0.00417		0.834	0.00255

*Note*: MES, BL, SJ, JT, JX, DX, HZ, ZQ, RY, SG, JLS, WGS, and PB represent wild populations, which were divided into three genetic lineages—YD (MES, BL, SJ, JT, JX), MD (DX, HZ, ZQ, RY, SG, JLS, WGS), and YK (PB)—based on the phylogenetic analysis of chlorotypes. *h*—haplotype diversity; π—nucleotide diversity. Values in parentheses indicate the frequency of each haplotype.

The results of network analysis were corroborated by the phylogenetic analysis of chlorotypes. The phylogenetic tree constructed using the ML method divided the seven chlorotypes into three genetic lineages (Figure 2d). Chlorotype C5 formed a separate clade (YK lineage); chlorotypes C1, C4, and C7 clustered together in the YD lineage; and chlorotypes C2, C3, and C6 formed the MD lineage. Total genetic diversity across all wild populations (*H*_T_ = 0.881) was higher than the average within‐population genetic diversity (*H*_S_ = 0.036), resulting in high population differentiation (*G*_ST_ = 0.959, *N*_ST_ = 0.997). A permutation test revealed a significant phylogeographic structure in *S. grosvenorii* (*N*_ST_ > *G*_ST_; *p* < .05). The results of overall AMOVA (Table 8) revealed that 99.44% of the molecular variation in cpDNA was found among the 13 populations, whereas 85.49% was found among the three cpDNA‐based phylogenetic lineages (YD, MD, and YK). The estimated divergence times of three cpDNA‐based lineages (YK vs. YD/MD and YD vs. MD) ranged from 1.35 to 1.60 Mya or from 4.07 to 4.79 Mya, assuming minimum and maximum synonymous substitution rates in cpDNA, respectively (Figure 2d).

**TABLE 8:** Analysis of molecular variance (AMOVA) of *S. grosvenorii* populations, based on cpDNA regions (*trn*R‐*atp*A, *trn*H‐*psb*A, and *trn*L‐*trn*F) and nuclear genes (*CHS* and *EDL2*)*.*

Lineage	Source of variation	Sequence variation (%)					
		cpDNA	*F*st	*CHS*	*F*st	*EDL2*	*F*st
All populations	Among populations	99.44	0.994	50.95	0.509	37.86	0.379
	Within populations	0.56		49.05		62.14	
Genetic lineages	Among lineages	85.49	0.996	8.45	0.526	1.19	0.403
	Among populations within a lineage	14.14		44.15		39.10	
	Within populations	0.37		47.40		59.71	

*Note*: All values are significant at *p* < .001. Sequence variation in cpDNA and nuclear genes was analyzed in all 13 wild populations of *S. grosvenorii* (MES, BL, SJ, JT, JX, DX, HZ, ZQ, RY, SG, JLS, WGS, PB) as one lineage and three genetic lineages (YD, MD, YK; identified based on the phylogenetic analysis of chlorotypes).

### 3.2 Population structure based on *CHS* and *EDL2* haplotypes

**FIGURE 3:**
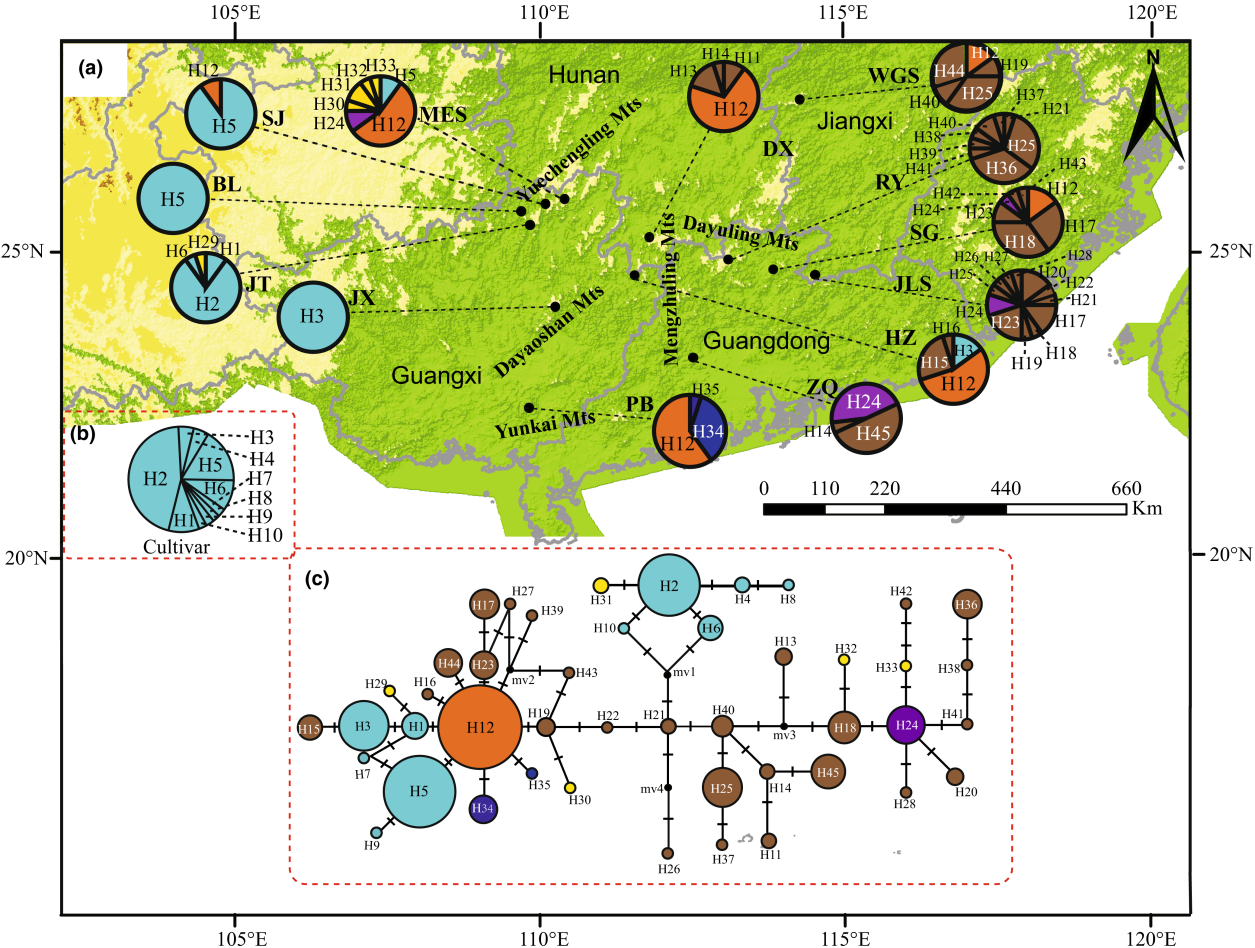
Geographical distribution, median‐joining network for 45 *CHS* haplotypes of *S. grosvenorii*. (a) *CHS* haplotypes of 13 wild populations of *S. grosvenorii*. (b) *CHS* haplotypes of 21 individuals of cultivated *S. grosvenorii*. (c) Median‐joining network of 45 *CHS* haplotypes resolved in *S. grosvenorii*. Haplotypes of cultivated *S. grosvenorii* are indicated in cyan color; the most widespread ancestral haplotype (H12) is indicated in orange color; haplotypes specific to the YD, MD, and YK lineages are indicated in yellow, brown, and dark blue, respectively; and haplotypes shared by YD and MD lineages are indicated in purple. Small solid circles with “mv” indicate unsampled or extinct haplotypes. Each black bar between the lines connecting the haplotypes represents one mutation.

The *CHS* sequence alignment was 1014 bp in length and contained 25 polymorphic sites (Table 5). Forty‐five *CHS* haplotypes (H1–H45) were identified, among which twenty‐five were unique to a single population. Haplotype H12 was shared by seven populations (MES, SJ, HZ, DX, SG, WGS, and PB), making it the most common and the most widely distributed haplotype (Figure 3a). Additionally, eight haplotypes (H1, H5, H16, H19, H23, H34, H35, and H44) were derived from haplotype H12 (Figure 3c), which indicated that H12 is one of the ancient haplotypes. Six haplotypes (H2, H4, H6, H8, H10, and H31) were found in two wild populations (MES and JT) and cultivars. Populations BL and JX contained only H5 and H3 haplotypes, respectively (Figure 3a, Table 7). The values of *h* and *π* in wild populations were 0.909 (range: 0–0.932) and 0.00406 (range: 0–0.00422), respectively (Table 7). The highest values of *h* (0.932) and *π* (0.00422) were found in the JLS and SG populations, respectively (Table 7).

**FIGURE 4:**
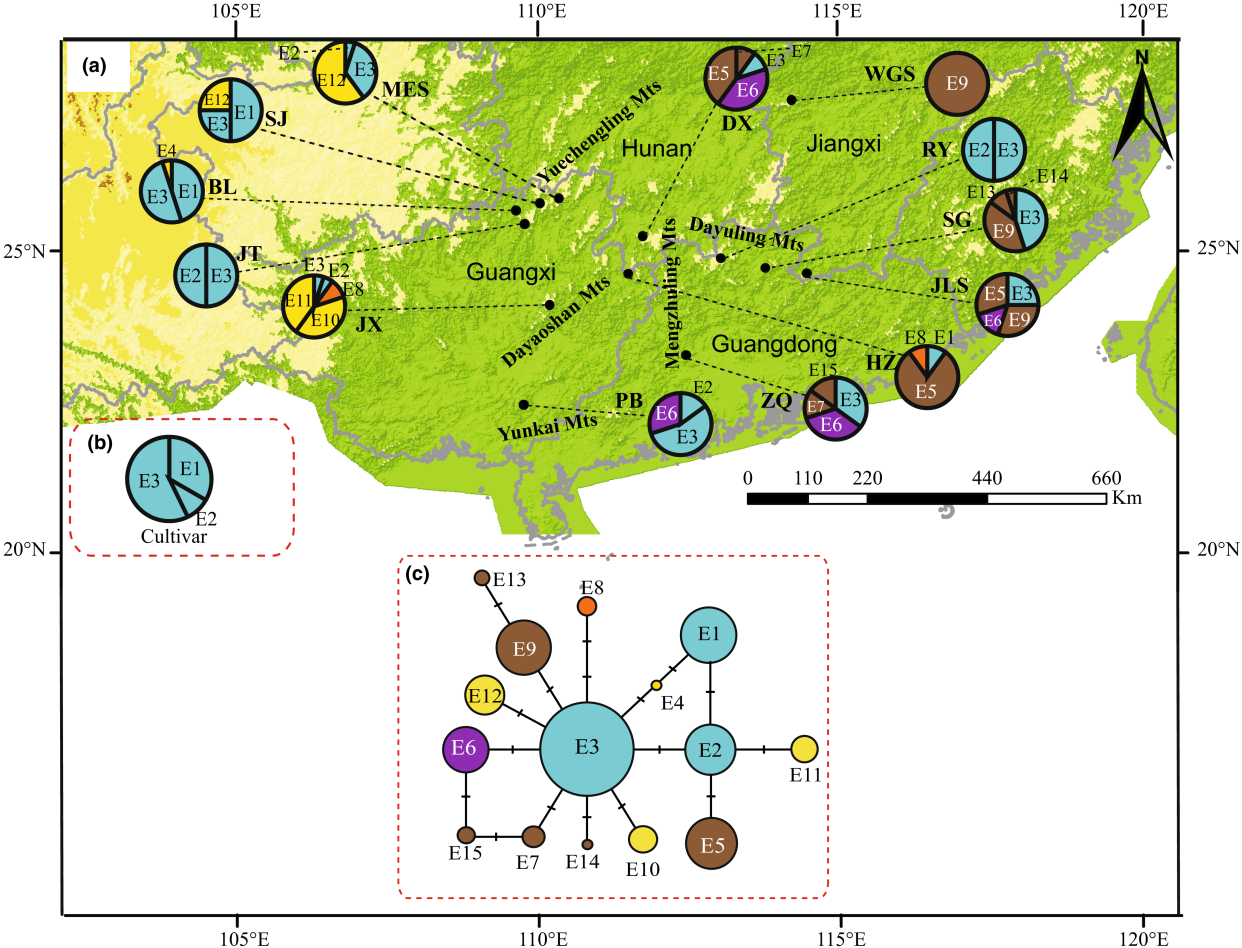
Geographical distribution and median‐joining network of 15 *EDL2* haplotypes of *S. grosvenorii*. (a) *EDL2* haplotypes of 13 wild populations of *S. grosvenorii*. (b) *EDL2* haplotypes of 21 individuals of cultivated *S. grosvenorii*. (c) Median‐joining network of 15 *EDL2* haplotypes resolved in *S. grosvenorii*. Haplotypes of cultivated *S. grosvenorii* are indicated in cyan color; haplotypes specific to the YD and MD lineages are indicated in yellow and brown, respectively; haplotypes shared by YD and MD lineages are indicated in orange; and haplotypes shared by YK and MD lineages are indicated in purple. Each black bar between the lines connecting the haplotypes represents one mutation.

The *EDL2* sequence alignment was 607 bp in length and contained 13 polymorphic sites, resulting in 15 haplotypes (E1–E15, Table 6). The *EDL2* haplotype network showed a “star‐like” topology, with haplotype E3 at the center (Figure 4c). Eight haplotypes (E2, E4, E6, E7, E9, E10, E12, and E14) were separated from haplotype E3 by one mutational step. In addition, E3 was the most common and the most widely distributed haplotype was found in all populations, except HZ and WGS. This suggests that E3 is likely the ancient haplotype. Six haplotypes (E4, E10, E11, E13, E14, and E15) were exclusive to a single population (Figure 4a, Table 7). The WGS population contained only the E9 haplotype. The values of *h* and *π* in wild populations inferred from the *EDL2* data were 0.854 (range: 0–0.774) and 0.00264 (range: 0–0.00315), respectively (Table 7). The highest values of *h* (0.774) and *π* (0.00315) were found in population JLS and JX, respectively (Table 7).

Similar to the results of cpDNA data analysis, the *H*_T_ of nuclear genes (0.965 for *CHS*, 0.904 for *EDL2*) was higher than the *H*_S_ of these genes (0.559 for *CHS*, 0.589 for *EDL2*). Although the *N*_ST_ value of *CHS* (0.464) was higher than the corresponding *G*_ST_ value (0.420), the *N*_ST_ value of *EDL2* (0.344) was lower than the corresponding *G*_ST_ value (0.349), the difference was not significant (*p* > .05), suggesting that the phylogeographic structure of *S. grosvenorii* based on the nuclear gene data was also not significant. The results of AMOVA (Table 8) showed that the variation in *CHS* sequence among populations (50.95%) was only slightly higher than that within populations (49.05%). In contrast to the results of cpDNA and *CHS* data analyses, the among‐population variation in *EDL2* (37.86%) was lower than the within‐population variation (62.14%) (Table 8). When the populations were divided into three chlorotype‐based genetic lineages, the sequence data of both *CHS* and *EDL2* showed a similar level of within‐population variation (47.40% for *CHS*, 59.71% for *EDL2*), but the among‐lineage variation in both *CHS* and *EDL2* was much lower (8.45% for *CHS*, 1.19% for *EDL2*) (Table 8).

### 3.3 Demographic history of *S. grosvenorii*

**FIGURE 5:**
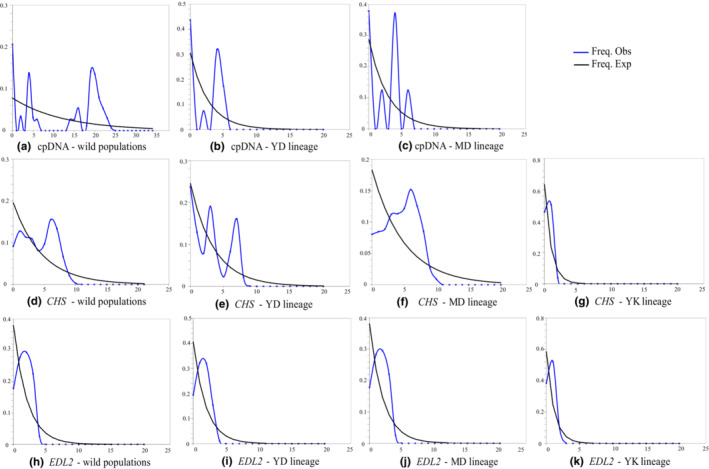
Mismatch distribution analysis of *S. grosvenorii* based on cpDNA and nuclear gene data. The blue line represents observed values whereas the black line shows expected values under a model of sudden (stepwise) population expansion. MD, YD, and YK represent the three genetic lineages identified by the phylogenetic analysis of chlorotypes.

The values of Tajima's *D*, Fu and Li's *D*, and Fu's *F*s for cpDNA haplotypes of the wild *S. grosvenorii* populations were positive (Table 9). In addition, the mismatch distribution was multimodal (Figure 5a), and both the SSD and *H*_Rag_ statistics were significant (Table 9), indicating that *S. grosvenorii* did not undergo population expansion. The resulting unimodal mismatch distribution detected by the *EDL2* data in *S. grosvenorii* suggested a model of sudden expansion (Figure 5h). This pattern was also indicated by the significant Fu's *Fs* value (−16.58458, *p* < .001), as well as the nonsignificant SSD and *H*_Rag_ statistics (*p* > .05) of *CHS*. However, the nonsignificant Tajima's *D* and Fu and Li's *D* values of the two nuclear genes and the significant SSD and *H*_Rag_ statistics of *EDL2* also rejected the hypothesis of rapid expansion (Table 9).

**TABLE 9:** Demographic expansion probabilities computed on the combined cpDNA and nuclear gene data of *S. grosvenorii.*

DNA	Lineage	Tajima's *D*	Fu and Li's *D*	Fu's *Fs*	SSD	*H*_Rag_
cpDNA	Wild populations	3.04911	2.05743*	23.03719	0.05629**	0.09460**
	YD lineage	2.52466	1.17469	5.55884	0.15034*	0.35340*
	MD lineage	2.37630*	1.14838	6.83365	0.15498*	0.48304**
	YK lineage	–	–	–	–	–
*CHS*	Wild populations	0.02792	−0.42074	−16.58458**	0.00844	0.01332
	YD lineage	1.16571	1.42189	−0.17043	1.11107	0.07240
	MD lineage	0.34464	0.00309	−8.97721*	0.00290	0.00772
	YK lineage	0.63250	−0.59347	0.06641	0.03222	0.22302
*EDL2*	Wild populations	−0.57491	0.77656	−3.99761	0.00970*	0.08172**
	YD lineage	0.19664	1.19344	−0.62963	0.00587**	0.06472**
	MD lineage	−0.23290	0.61231	−1.86843	0.00749	0.05564
	YK lineage	0.61051	0.86615	0.47455	0.31690	0.20945

*Note*: *H*_Rag_—raggedness index. Values not marked with asterisks were not significant. The analysis could not be performed on the YK lineage, which contained only one cpDNA‐based haplotype.

Abbreviation: SSD, sum of squared deviations.

* *p* < .05

** *p* < .001.

When the test was performed on the three chlorotype‐based genetic lineages identified in this study, the Tajima's *D* values of MD lineage in cpDNA data were significant and positive (2.37630, *p* < .05); the Fu's *Fs* values of MD lineage in *CHS* data were significant and negative (−8.97721, *p* < .05); and the remaining Tajima's *D*, Fu and Li's *D*, and Fu's *F*s values were nonsignificant (Table 9). The SSD and *H*_Rag_ statistics of the three lineages in *CHS* data and those of MD and YK lineages in *EDL2* data were nonsignificant (*p* > .05) (Table 9). Unimodal mismatch distribution was detected in the three lineages based on the *EDL2* data (Figure 5i–k) and in the YK lineages based on the *CHS* data (Figure 5g). Multimodal mismatch distribution was found in the YD and MD lineages based on both cpDNA and *CHS* data (Figure 5b,c,e,f).

## 4 DISCUSSION

### 4.1 Genetic structure and phylogeographic history of *S. grosvenorii*

Our cpDNA results showed that all haplotypes of *S. grosvenorii* were split into three phylogenetic lineages (Figure 2d). Significantly, the three divergent lineages of *S. grosvenorii* were confined to different mountain ranges, showing a clear‐cut geographic structure (Figure 2a,d). This clear geographical structuring was also supported by AMOVA results (Table 8), which indicated that 85.49% of the genetic variation in cpDNA could be attributed to variation among regions. Specifically, the first lineage, which included the MES, BL, SJ, JT, and JX populations, was distributed in Yuechengling‐Dayaoshan Mountains (in the western range of Nanling Mountains), which was represented by chlorotypes C1, C4, and C7. The HZ, DX, ZQ, JLS, RY, SG, and WGS populations, which comprised the second lineage (C2, C3, and C6), were distributed in Mengzhuling‐Dayuling Mountains (in the central and eastern Nanling Mountains). The third lineage (C5), which contained only the PB population, was found in Yunkai Mountains. The present distribution of chlorotypes leads us to propose that species distribution was fragmented into independent geographical regions in the past, resulting in physical separation among the different mountains. This hypothesis was supported by the detection of unique chlorotypes in different geographic regions, a large number of mutational steps among three main divergent lineages in the network (at least 14 mutational steps between the MD and YK lineages, 19 between MD and YD, and 16 between YK and YD; Figure 2c), and strong plastid phylogeographic structure. The divergence among the major cpDNA lineages of *S. grosvenorii* is estimated to have occurred between the early Pliocene (5.33 to 3.60 Mya) and early Pleistocene (2.58 to 0.80 Mya) (Figure 2d), implying that *S. grosvenorii* survived the extensive climate changes occurring during almost the entire Quaternary. Mismatch distribution analysis and neutrality tests of our cpDNA data (Table 9, Figure 5) revealed no recent population expansion for the whole species, as well as for the haplotype groups from different mountain regions. We thus propose that *S. grosvenorii* could have survived in situ through glacial periods. This finding is consistent with the pronounced phylogeographic structure reported for many plant species in subtropical China that probably resulted from long‐term survival in multiple glacial refugia and limited inter/postglacial expansions (Lei et al., 2012; Lopez‐Pujol et al., 2011; Qiu et al., 2011; Zhang et al., 2013). Mountains have generally provided refuge for species because their topographical diversity allows enough eco‐environmental stability, buffering them against extreme climatic fluctuations (Feliner, 2014; López‐Pujol et al., 2011; Spehn et al., 2010). The Nanling and Yunkai Mountains, which are characterized by a rugged topography, are well recognized as refugia for many other subtropical species (Qiu et al., 2011), including not only the long‐lived or relict tree species such as *Eurycorymbus cavaleriei* (Wang et al., 2009) and *Cathaya argyrophylla* (Feliner, 2014) but also the perennial herb *Eomecon chionantha* (Tian et al., 2018) and herbaceous climber *Tetrastigma hemsleyanum* (Wang et al., 2015).

Haplotypes shared among the three lineages (e.g., haplotypes H12, E3, and E2) represent the internal nodes of the network, could be considered as ancestral, and would predate the divergence of populations. We did not find a firm recent population expansion signal in our nuclear dataset. The distribution of these haplotypes across all regions could probably be attributed more to the persistence of ancestral polymorphisms than to recent gene flow and dispersal. Thus, the most likely scenario is that *S. grosvenorii* experienced an ancient expansion event that brought ancestral haplotypes H12 and E3 to different parts of the range, which resulted in the origin of numerous derived haplotypes in these isolated locations; for example, 25 *CHS* haplotypes and 6 *EDL2* haplotypes, which were private to a single population. Nevertheless, the nuclear haplotypes H3, E1, and E8 occurred in MD (HZ population only) and YD lineages, and E6 was shared between the MD and YK lineages. These results suggest limited pollen dispersal among lineages within *S. grosvenorii*.

### 4.2 Origin of cultivated *S. grosvenorii* and implications for improvement and conservation of genetic resources

Population genetics‐based insights will enable a better understanding of the origin of the crop and will help to identify raw materials for breeding and crop improvement (Ramanatha & Hodgkin, 2002; Turner‐Hissong et al., 2020). *S. grosvenorii* is documented to have been cultivated in Longsheng, Yongfu, and Lingui counties of Guilin for at least 140 years (Ding et al., 2015; Yang, 2004) and then was introduced from Guangxi to Guangdong, Hunan, Jiangxi, and Guizhou Provinces (Li et al., 2004). Our data revealed the presence of only one chlorotype (C1) in cultivated *S. grosvenorii* (Figure 2b), which clearly suggests that *S. grosvenorii* was most likely domesticated from a single center of origin. Interestingly, populations MES, BL, and JT from Guilin shared the same chlorotype (C1) and several nuclear haplotypes with cultivars, indicating that the main cultivars currently grown in Yongfu, Lingui, and Longsheng originated from wild populations distributed in Guilin, Guangxi, consistent with the principals of “nearby domestication.” Indeed, the initial cultivation of *S. grosvenorii* followed traditional practices, whereby plants were directly collected from the local wild resources and then cultivated in the field (Li et al., 2004).

The populations of MD lineage showed higher genetic diversity, and the populations of YD and YK lineages also exhibited a certain level of genetic diversity. Although wild populations of *S. grosvenorii* are believed to possess abundant genetic variation (*H*_T_ = 0.881 for cpDNA; *H*_T_ = 0.965 for *CHS*; and *H*_T_ = 0.904 for *EDL*2), it is obvious that gene‐rich pools of wild relatives have not been exploited for the improvement of cultivars. The three genetic lineages identified in this study contained a large number of unique haplotypes (Figures 3a and 4a, Table 7). This unique composition of the genetic lineages shows a great potential for improving the cultivated varieties. However, during the fieldwork, we noted that wild populations of *S. grosvenorii* had suffered a rapid decline and were even extirpated at some distribution points, because of habitat deterioration. Establishing a germplasm bank for *S. grosvenorii* is critical for conservation purposes, and germplasm collections should be exhaustive to compensate for the unique genetic compositions. Meanwhile, only one population (PB) is found in the Yunkai Mountains and therefore deserves particular attention as a prime ex situ conservation target. Additionally, we propose that populations from the MD lineage and YD lineage (the geographical origin of cultivars) should be utilized as the plant material or core gene pool for breeding and crop improvement, for which both the in situ and ex situ methods should be adopted.

## 5 CONCLUSIONS

The molecular data collected in the current study permit the first phylogeographic analysis of *S. grosvenorii* distributed in subtropical China. Our cpDNA results showed that three main lineages of the present populations of *S. grosvenorii* occupy different mountain ranges and have been isolated from each other for a long period of time. *S. grosvenorii* likely experienced an ancient range expansion and survived in multiple refuges during the glacial periods, resulting in fragmentation in different mountainous areas. *S. grosvenorii* cultivars originated from wild populations MES, BL, and JT in Guilin, Guangxi, consistent with the principle of nearby domestication. This phylogeographical study also provides a genetic basis for the utilization and conservation of *S. grosvenorii* germplasm.

## AUTHOR CONTRIBUTIONS

**Bingbin Xie:** Conceptualization (equal); data curation (lead); formal analysis (lead); investigation (equal); methodology (lead); software (lead). **Bowen Lai:** Conceptualization (equal); data curation (supporting); formal analysis (supporting); writing – original draft (lead); writing – review and editing (supporting). **Liping Chen:** Data curation (supporting); formal analysis (supporting); investigation (equal); methodology (supporting); software (supporting). **Sujuan Wei:** Conceptualization (equal); methodology (supporting); supervision (supporting); validation (supporting); writing – review and editing (equal). **Shaoqing Tang:** Conceptualization (equal); funding acquisition (lead); project administration (lead); supervision (lead); validation (lead); visualization (lead); writing – review and editing (equal).

## CONFLICT OF INTEREST STATEMENT

The author declares that they have no conflict of interest.

## Data Availability

The data that support the findings of this study are openly available in the NCBI Nucleotide database at https://www.ncbi.nlm.nih.gov/nuccore/, under accession number: MK356960–MK356967, MK356969–MK356970, MK356972–MK357025, OQ134869–OQ134871.

## References

[ece310181-bib-0001] Abbott, R. J. , Smith, L. C. , Milne, R. I. , Crawford, R. M. M. , Wolff, K. , & Balfour, J. (2000). Molecular analysis of plant migration and refugia in the Arctic. Science, 289(5483), 1343–1346. 10958779 10.1126/science.289.5483.1343

[ece310181-bib-0068] An, M. , Zeng, L. Y. , Zhang, T. C. , & Zhong, Y. (2015). Phylogeography of *Thlaspi arvense* (Brassicaceae) in China inferred from chloroplast and nuclear DNA sequences and ecological niche modeling. International Journal of Molecular Sciences, 16(6), 13339–13355. 26110380 10.3390/ijms160613339 PMC4490498

[ece310181-bib-0002] Avise, J. C. (2000). Phylogeography: The history and formation of species. Harvard University Press.

[ece310181-bib-0003] Bandelt, H. J. , Forster, P. , & Rohl, A. (1999). Median‐joining networks for inferring intraspecific phylogenies. Molecular Biology and Evolution, 16(1), 37–48. 10331250 10.1093/oxfordjournals.molbev.a026036

[ece310181-bib-0004] Caicedo, A. L. , & Schaal, B. A. (2004). Population structure and phylogeography of *Solanum pimpinellifolium* inferred from a nuclear gene. Molecular Ecology, 13(7), 1871–1882. 15189210 10.1111/j.1365-294X.2004.02191.x

[ece310181-bib-0005] Chinese Pharmacopoeia Commission . (2015). Pharmacopoeia of the People's Republic of China, Volume I. China Medical Science Press.

[ece310181-bib-0007] Clark, A. G. (1990). Inference of haplotypes from PCR‐amplified samples of diploid populations. Molecular Biology and Evolution, 7(2), 111–122. 2108305 10.1093/oxfordjournals.molbev.a040591

[ece310181-bib-0008] Dane, F. , & Lang, P. (2004). Sequence variation at cpDNA regions of watermelon and related wild species: Implications for the evolution of *Citrullus* haplotypes. American Journal of Botany, 91(11), 1922–1929. 21652338 10.3732/ajb.91.11.1922

[ece310181-bib-0009] Day‐Rubenstein, K. A. , Heisey, P. W. , Shoemaker, R. A. , Sullivan, J. , & Frisvold, G. B. (2005). Crop genetic resources: An economic appraisal. Economic Information Bulletin, 2.

[ece310181-bib-0010] Ding, T. , Su, T. , & Liu, J. L. (2015). Status，problems and countermeasures of industrial development of *Momordica Grosvenori* Swingle in North Guangxi. Journal of Anhui Agricultural Sciences, 43(35), 326–327+335.

[ece310181-bib-0011] Doyle, J. J. , & Doyle, J. L. (1987). A rapid DNA isolation procedure for small quantities of fresh leaf tissue. Phyotochemical Bulletin, 19, 11–15.

[ece310181-bib-0012] Drummond, A. J. , & Rambaut, A. (2007). BEAST: Bayesian evolutionary analysis by sampling trees. BMC Evolutionary Biology, 7(1), 1–8. 17996036 10.1186/1471-2148-7-214 PMC2247476

[ece310181-bib-0013] Excoffier, L. , Laval, G. , & Schneider, S. (2005). Arlequin (version 3.0): An integrated software package for population genetics data analysis. Evolutionary Bioinformatics, 1(4), 47–50. PMC2658868 19325852

[ece310181-bib-0014] Feliner, G. N. (2014). Patterns and processes in plant phylogeography in the Mediterranean Basin. A review. Perspectives in Plant Ecology, Evolution and Systematics, 16(5), 265–278.

[ece310181-bib-0015] Fu, Y. X. (1997). Statistical tests of neutrality of mutations against population growth, hitchhiking and background selection. Genetics, 147(2), 915–925. 9335623 10.1093/genetics/147.2.915 PMC1208208

[ece310181-bib-0016] Fu, Y. X. , & Li, W. H. (1993). Statistical tests of neutrality of mutations. Genetics, 133(3), 693–709. 8454210 10.1093/genetics/133.3.693 PMC1205353

[ece310181-bib-0069] Gong, W. , Liu, W. Z. , Gu, L. , Kaneko, S. G. , Koch, M. A. , & Zhang, D. X. (2016). From glacial refugia to wide distribution range: demographic expansion of *Loropetalum chinense* (Hamamelidaceae) in Chinese subtropical evergreen broadleaved forest. Organisms Diversity and Evolution, 16(1), 23–38.

[ece310181-bib-0017] Guo, J. , Xu, W. B. , Hu, Y. , Huang, J. , Zhao, Y. Y. , Zhang, L. , Huang, C. H. , & Ma, H. (2020). Phylotranscriptomics in Cucurbitaceae reveal multiple whole‐genome duplications and key morphological and molecular innovations. Molecular Plant, 13(8), 1117–1133. 32445889 10.1016/j.molp.2020.05.011

[ece310181-bib-0018] Hall, T. A. (1999). BioEdit: A user‐friendly biological sequence alignment editor and analysis program for windows 95/98/NT. Nucleic Acids Symposium Series, 41(41), 95–98.

[ece310181-bib-0019] Ikeda, H. , Fujii, N. , & Setoguchi, H. (2009). Application of the isolation with migration model demonstrates the Pleistocene origin of geographic differentiation in Cardamine nipponica (Brassicaceae), an endemic Japanese alpine plant. Molecular Biology and Evolution, 26(10), 2207–2216. 19567916 10.1093/molbev/msp128

[ece310181-bib-0020] Kalyaanamoorthy, S. , Minh, B. Q. , Wong, T. K. , Von Haeseler, A. , & Jermiin, L. S. (2017). ModelFinder: Fast model selection for accurate phylogenetic estimates. Nature Methods, 14(6), 587–589. 28481363 10.1038/nmeth.4285 PMC5453245

[ece310181-bib-0021] Kasai, R. , Nie, R. L. , Nashi, K. , Ohtani, K. , Zhou, J. , Tao, G. D. , & Tanaka, O. (1989). Sweet cucurbitane glycosides from fruits of *Siraitia siamensis* (chi‐zi luo‐han‐guo), a Chinese folk medicine. Agricultural and Biological Chemistry, 53(12), 3347–3349.

[ece310181-bib-0022] Kumar, S. , Stecher, G. , & Tamura, K. (2016). MEGA7: Molecular evolutionary genetics analysis version 7.0 for bigger datasets. Molecular Biology and Evolution, 33(7), 1870–1874. 27004904 10.1093/molbev/msw054 PMC8210823

[ece310181-bib-0023] Lei, M. , Wang, Q. , Wu, Z. J. , López‐Pujol, J. , Li, D. Z. , & Zhang, Z. Y. (2012). Molecular phylogeography of *Fagus engleriana* (Fagaceae) in subtropical China: Limited admixture among multiple refugia. Tree Genentic and Genomes, 8(6), 1203–1212.

[ece310181-bib-0024] Li, F. , Li, D. P. , Jiang, S. Y. , & Zhang, H. R. (2004). Cultivation, development and utilization of Luo Han Guo. China Forestry Publishing House.

[ece310181-bib-0025] Li, J. Q. (1993). A revision of the genus *Siraitia* Merr. And two new genera of cucurbitaceae. Acta Phytotaxonomica Sinica, 31(1), 45–55.

[ece310181-bib-0026] Liao, P. C. , Kuo, D. C. , Lin, C. C. , Ho, K. C. , Lin, T. P. , & Hwang, S. Y. (2010). Historical spatial range expansion and a very recent bottleneck of Cinnamomum kanehiraeHay. (Lauraceae) in Taiwan inferred from nuclear genes. BMC Evolutionary Biology, 10(1), 124. 20433752 10.1186/1471-2148-10-124 PMC2880300

[ece310181-bib-0027] Librado, P. , & Rozas, J. (2009). DnaSP v5: A software for comprehensive analysis of DNA polymorphism data. Bioinformatics, 25(11), 1451–1452. 19346325 10.1093/bioinformatics/btp187

[ece310181-bib-0028] Lopez‐Pujol, J. , Zhang, F. M. , Sun, H. Q. , Ying, T. S. , & Ge, S. (2011). Mountains of southern China as “plant museums” and “plant cradles”: Evolutionary and conservation insights. Mountain Research and Development, 31(3), 261–269.

[ece310181-bib-0029] López‐Pujol, J. , Zhang, F. M. , Sun, H. Q. , Ying, T. S. , & Ge, S. (2011). Centres of plant endemism in China: Places for survival or for speciation? Journal of Biogeography, 38(7), 1267–1280.

[ece310181-bib-0030] Lu, Y. , Qin, S. Y. , Hu, T. T. , & Wei, Z. X. (2022). Create a ten billion “sweet industry” in Guangxi. Sci‐Tech and Development of Enterprise, 2, 25–27.

[ece310181-bib-0031] Nguyen, L. T. , Schmidt, H. A. , Von Haeseler, A. , & Minh, B. Q. (2015). IQ‐TREE: A fast and effective stochastic algorithm for estimating maximum‐likelihood phylogenies. Molecular Biology and Evolution, 32(1), 268–274. 25371430 10.1093/molbev/msu300 PMC4271533

[ece310181-bib-0032] Ni, J. , Yu, G. , Harrison, S. P. , & Prentice, I. C. (2010). Palaeovegetation in China during the late quaternary: Biome reconstructions based on a global scheme of plant functional types. Palaeogeography, Palaeoclimatology, Palaeoecology, 289(1), 44–61.

[ece310181-bib-0033] Pandey, A. K. , & Chauhan, O. P. (2019). Monk fruit (*Siraitia grosvenorii*)‐health aspects and food applications. Pantnagar Journal of Research, 17(3), 191–198.

[ece310181-bib-0034] Peng, Y. T. , Tang, S. Q. , Li, B. L. , & Liu, Y. H. (2005). Genetic diversity of *Siraitia grosvenorii* detected by ISSR markers. Biodiversity Science, 13(1), 36–42.

[ece310181-bib-0035] Pons, O. , & Petit, R. (1996). Measuring and testing genetic differentiation with ordered versus unordered alleles. Genetics, 144(3), 1237–1245. 8913764 10.1093/genetics/144.3.1237 PMC1207615

[ece310181-bib-0036] Prescott‐Allen, R. , & Prescott‐Allen, C. (2013). Genes from the wild: Using wild genetic resources for food and raw materials. Routledge.

[ece310181-bib-0037] Qian, H. , & Ricklefs, R. E. (2000). Large‐scale processes and the Asian bias in species diversity of temperate plants. Nature, 407(6801), 180–182. 11001054 10.1038/35025052

[ece310181-bib-0038] Qiu, Y. X. , Fu, C. X. , & Comes, H. P. (2011). Plant molecular phylogeography in China and adjacent regions: Tracing the genetic imprints of quaternary climate and environmental change in the world's most diverse temperate flora. Molecular Phylogenetics and Evolution, 59(1), 225–244. 21292014 10.1016/j.ympev.2011.01.012

[ece310181-bib-0039] Ramanatha, R. V. , & Hodgkin, T. (2002). Genetic diversity and conservation and utilization of plant genetic resources. Plant Cell, Tissue and Organ Culture, 68(1), 1–19.

[ece310181-bib-0040] Rambaut, A. , Drummond, A. J. , Xie, D. , Baele, G. , & Suchard, M. A. (2018). Posterior summarization in Bayesian phylogenetics using tracer 1.7. Systematic Biology, 67(5), 901–904. 29718447 10.1093/sysbio/syy032 PMC6101584

[ece310181-bib-0041] Rogers, A. R. , & Harpending, H. (1992). Population growth makes waves in the distribution of pairwise genetic differences. Molecular Biology and Evolution, 9(3), 552–569. 1316531 10.1093/oxfordjournals.molbev.a040727

[ece310181-bib-0042] Sang, T. , Crawford, D. J. , & Stuessy, T. F. (1997). Chloroplast DNA phylogeny, reticulate evolution, and biogeography of *Paeonia* (Paeoniaceae). American Journal of Botany, 84(8), 1120–1136. 21708667

[ece310181-bib-0044] Shi, Y. F. , Cui, Z. J. , & Su, Z. (2006). The quaternary glaciations and environmental variations in China. Hebei Science and Technology Press.

[ece310181-bib-0046] Spehn, E. M. , Rudmann‐Maurer, K. , Korner, C. , & Maselli, D. (2010). Mountain biodiversity and global change. GMBA‐DIVERSITAS.

[ece310181-bib-0047] Taberlet, P. , Gielly, L. , Pautou, G. , & Bouvet, J. (1991). Universal primers for amplification of three non‐coding regions of chloroplast DNA. Plant Molecular Biology, 17(5), 1105–1109. 1932684 10.1007/BF00037152

[ece310181-bib-0048] Tajima, F. (1989). Statistical method for testing the neutral mutation hypothesis by DNA polymorphism. Genetics, 123(3), 585–595. 2513255 10.1093/genetics/123.3.585 PMC1203831

[ece310181-bib-0049] Tang, S. Q. , Bin, X. Y. , Peng, Y. T. , Zhou, J. Y. , Wang, L. , & Zhong, Y. (2007). Assessment of genetic diversity in cultivars and wild accessions of Luohanguo (*Siraitia grosvenorii* [Swingle] AM Lu et ZY Zhang), a species with edible and medicinal sweet fruits endemic to southern China, using RAPD and AFLP markers. Genetic Resources and Crop Evolution, 54(5), 1053–1061.

[ece310181-bib-0050] Tang, S. Q. , Li, Y. , Geng, Y. , Zhang, G. R. , Wang, L. , & Zhong, Y. (2007). Clonal and spatial genetic structure in natural populations of Luohanguo (*Siraitia grosvenorii*), an economic species endemic to South China, as revealed by RAPD markers. Biochemical Systematics and Ecology, 35(9), 557–565.

[ece310181-bib-0051] Thompson, J. D. , Higgins, D. G. , & Gibson, T. J. (1994). CLUSTAL W: Improving the sensitivity of progressive multiple sequence alignment through sequence weighting, position‐specific gap penalties and weight matrix choice. Nucleic Acids Research, 22(22), 4673–4680. 7984417 10.1093/nar/22.22.4673 PMC308517

[ece310181-bib-0052] Tian, S. , Kou, Y. X. , Zhang, Z. R. , Yuan, L. , Li, D. R. , López‐Pujol, J. , Fan, D. M. , & Zhang, Z. Y. (2018). Phylogeography of *Eomecon chionantha* in subtropical China: The dual roles of the Nanling Mountains as a glacial refugium and a dispersal corridor. BMC Evolutionary Biology, 18(1), 1–12. 29426277 10.1186/s12862-017-1093-x PMC5807764

[ece310181-bib-0053] Turner‐Hissong, S. D. , Mabry, M. E. , Beissinger, T. M. , Ross‐Ibarra, J. , & Pires, J. C. (2020). Evolutionary insights into plant breeding. Current Opinion in Plant Biology, 54, 93–100. 32325397 10.1016/j.pbi.2020.03.003

[ece310181-bib-0054] Wang, J. , Gao, P. X. , Kang, M. , Lowe, A. J. , & Huang, H. W. (2009). Refugia within refugia: The case study of a canopy tree (*Eurycorymbus cavaleriei*) in subtropical China. Journal of Biogeography, 36(11), 2156–2164.

[ece310181-bib-0055] Wang, S. Y. , Lu, H. Y. , Han, J. T. , Chu, G. Q. , Liu, J. Q. , & Negendank, J. F. W. (2012). Palaeovegetation and palaeoclimate in low‐latitude southern China during the last glacial maximum. Quaternary International, 248, 79–85.

[ece310181-bib-0056] Wang, X. M. , Feng, L. , Zhou, T. , Ruhsam, M. , Huang, L. , Hou, X. Q. , Sun, X. J. , Fan, K. , Huang, M. , Zhou, Y. , & Song, J. (2018). Genetic and chemical differentiation characterizes top‐geoherb and non‐top‐geoherb areas in the TCM herb rhubarb. Scientific Reports, 8(1), 9424. 29930263 10.1038/s41598-018-27510-1 PMC6013459

[ece310181-bib-0057] Wang, Y. H. , Jiang, W. M. , Comes, H. P. , Hu, F. S. , Qiu, Y. X. , & Fu, C. X. (2015). Molecular phylogeography and ecological niche modelling of a widespread herbaceous climber, Tetrastigma hemsleyanum (Vitaceae): Insights into Plio–Pleistocene range dynamics of evergreen forest in subtropical China. New Phytologist, 206(2), 852–867. 25639152 10.1111/nph.13261

[ece310181-bib-0058] Wolfe, K. H. , Li, W. H. , & Sharp, P. M. (1987). Rates of nucleotide substitution vary greatly among plant mitochondrial, chloroplast, and nuclear DNAs. Proceedings of the National Academy of Sciences, 84(24), 9054–9058. 10.1073/pnas.84.24.9054 PMC299690 3480529

[ece310181-bib-0059] Xia, M. , Han, X. , He, H. , Yu, R. B. , Zhen, G. , Jia, X. P. , Cheng, B. J. , & Deng, X. W. (2018). Improved de novo genome assembly and analysis of the Chinese cucurbit *Siraitia grosvenorii*, also known as monk fruit or luo‐han‐guo. GigaScience, 7(6), giy067. 29893829 10.1093/gigascience/giy067 PMC6007378

[ece310181-bib-0060] Yan, X. , Rivero‐Huguet, M. E. , Hughes, B. H. , & Marshall, W. D. (2008). Isolation of the sweet components from *Siraitia grosvenorii* . Food Chemistry, 107(3), 1022–1028.

[ece310181-bib-0061] Yang, M. Q. (2004). Germplasm resources and production advice of Luohanguo in Longsheng County. Southern Horticulture, 5, 19–20.

[ece310181-bib-0062] Zhang, D. , Gao, F. L. , Jakovlić, I. , Zou, H. , Zhang, J. , Li, W. X. , & Wang, G. T. (2020). PhyloSuite: An integrated and scalable desktop platform for streamlined molecular sequence data management and evolutionary phylogenetics studies. Molecular Ecology Resources, 20(1), 348–355. 31599058 10.1111/1755-0998.13096

[ece310181-bib-0070] Zhang, L. , Sun, F. F. , Ma, S. M. , Wang, C. C. , Wei, B. , & Zhang, Y. L. (2022). Phylogeography of *Amygdalus mongolica* in relation to Quaternary climatic aridification and oscillations in northwestern China. PeerJ, 10, e13345. 35509965 10.7717/peerj.13345 PMC9059755

[ece310181-bib-0063] Zhang, Z. Y. , Wu, R. , Wang, Q. , Zhang, Z. R. , López‐Pujol, J. , Fan, D. M. , & Li, D. Z. (2013). Comparative phylogeography of two sympatric beeches in subtropical China: Species‐specific geographic mosaic of lineages. Ecology and Evolution, 3(13), 4461–4472. 24340187 10.1002/ece3.829 PMC3856746

[ece310181-bib-0064] Zhao, S. Q. , Chao, S. C. , & Salter, C. L. (1986). Physical geography of China. John Wiley & Sons Incorporated.

[ece310181-bib-0065] Zhou, J. Y. , & Tang, S. Q. (2006). Genetic diversity of cultivated Luohanguo (*Siraitia grosvenorii*) revealed by RAPD markers. Molecular Plant Breeding, 1, 71–78.

[ece310181-bib-0066] Zhou, J. Y. , Tang, S. Q. , Xiang, W. S. , & Bin, X. Y. (2005). Genetic diversity of cultivated Luohanguo (*Siraitia grosvenorii*) based on ISSR marker. Guihaia, 5, 431–436+503.

[ece310181-bib-0067] Zuckerkandl, E. , & Pauling, L. (1965). Evolutionary divergence and convergence in proteins. In V. Bryson & H. J. Vogel (Eds.), Evolving genes and proteins (pp. 97–166). Elsevier.

